# P53 nuclear stabilization is associated with *FHIT* loss and younger age of onset in squamous cell carcinoma of oral tongue

**DOI:** 10.1186/1472-6890-14-37

**Published:** 2014-08-09

**Authors:** Raju SR Adduri, Viswakalyan Kotapalli, Neha A Gupta, Swarnalata Gowrishankar, Mukta Srinivasulu, Mohammed Mujtaba Ali, Subramanyeshwar Rao, Shantveer G Uppin, Umanath K Nayak, Snehalatha Dhagam, Mohana Vamsy Chigurupati, Murali Dharan Bashyam

**Affiliations:** 1 Laboratory of Molecular Oncology, Centre for DNA Fingerprinting and Diagnostics, Nampally, Hyderabad 500001, India; 2 Apollo Hospitals, Jubilee Hills, Hyderabad India; 3 MNJ Institute of Oncology & Regional Cancer Centre, Red Hills, Hyderabad India; 4 Nizam’s Institute of Medical Sciences, Punjagutta, Hyderabad India; 5 Omega Hospitals, Jubilee Hills, Hyderabad, India; 6 Currently at National Centre for Cell Science, Ganeshkhind, Pune, India; 7 Currently at Basavatarakam Indo American Cancer Hospital & Research Institute, Hyderabad, India

**Keywords:** Oral tongue cancer, TP53, *FHIT*, EGFR, Disease specific survival

## Abstract

**Background:**

Squamous cell carcinoma of tongue (SCCT) is expected to harbor unique clinico-pathological and molecular genetic features since a significant proportion of patients are young and exhibit no association with tobacco or alcohol.

**Methods:**

We determined P53, epidermal growth factor receptor, microsatellite instability, human papilloma virus infection and loss of heterozygosity status at several tumor suppressor loci in one hundred and twenty one oral SCCT (SSCOT) samples and analyzed their association with clinico-pathological features and patient survival.

**Results:**

Our results revealed a significantly higher incidence of p53 nuclear stabilization in early (as against late) onset SCCOT. *FHIT* loss was significantly associated with p53 nuclear stabilization and the association was stronger in patients with no history of tobacco use. Samples harboring mutation in p53 DNA binding domain or exhibiting p53 nuclear stabilization, were significantly associated with poor survival.

**Conclusion:**

Our study has therefore identified distinct features in SCCOT tumorigenesis with respect to age and tobacco exposure and revealed possible prognostic utility of p53.

## Background

Squamous cell carcinoma of tongue (SCCT) is believed to be associated with late onset and tobacco use similar to other Head and neck squamous cell carcinoma (HNSCC) subtypes. An increased incidence in the young [1] and in individuals with no history of smoking and alcohol consumption [2] is reported for squamous cell carcinoma of oral tongue (SCCOT). SCCOT has the highest burden of young patients among all HNSCC subtypes and a significant proportion of patients belonging to this age group appear to include non-smokers [3]. In addition, young patients with SCCOT have frequent loco-regional recurrence [4] and poor prognosis [3]. Despite advances in cancer therapy, SCCOT five year survival rate has not improved in the last few decades [5]. All these factors make SCCOT a unique HNSCC subtype and yet molecular genetic studies designed specifically for this important cancer have been rare; most studies have been restricted to a single prognostic marker and/or a small cohort of patients [6].

We have conducted a retrospective study involving comprehensive molecular genetic and clinico-pathological analyses of one hundred and twenty one SCCOT samples; results revealed significant association of p53 nuclear stabilization with age of onset, *FHIT* loss and survival.

## Methods

### Patient samples

Previously untreated, surgically resected primary SCCOT specimens were collected from three hospitals in Hyderabad, India following informed consent and approval from respective hospital ethics committees (Institutional Ethics Committee of MNJ Institute of Oncology & Regional Cancer Centre, Institutional Ethics Committee of Apollo Hospitals and Ethics Committee of Omega Hospitals), as per modified Helsinki declaration of 2008 (http://www.wma.net/en/30publications/10policies/b3/). The study included a total of 121 tumor/normal sample pairs (all oral tongue; 106 freshly resected and 15 archived); all samples were from patients not associated with family history for any cancer. Median age of patients was 50 years with a male to female ratio of 2.0. Patients aged ≤45 years were considered as ‘young’ where as those aged ≥46 were considered as ‘old’. Surgically resected fresh tumor and matched normal tissues were collected in liquid nitrogen and preserved at −70°C after collecting representative pieces in buffered formalin for embedding in paraffin. 4 μM sections from tumor and matched normal formalin fixed and paraffin embedded (FFPE) blocks for each sample were stained with hematoxylin and eosin (H&E) to evaluate grade and absence of tumor infiltration, respectively. Clinical data and information pertaining to use of tobacco, alcohol and family history were obtained via personal interview in the form of questionnaire or from hospital medical records. Majority of tumors were well differentiated (86/121; 71.07%). Clinico-pathological details of the patient samples are given in Additional file 1: Table S1.

### Immunohistochemistry (IHC)

IHC was performed as per standard protocols [7] on tissues embedded into FFPE blocks mentioned above, as per standard practice though we are aware that this slice of tissue may not represent the whole tumor. 4 μM tumor sections were deparafinized and rehydrated in graded series of alcohol followed by heat induced epitope retrieval in citrate buffer at pH 6.0 (for p53) or proteinase K pretreatment (for epidermal growth factor receptor (EGFR)) and subjected to peroxidase quenching using 0.6% hydrogen peroxide in methanol. Sections were incubated with 1 μg/ml anti-p53 (DO-1, EMD Millipore Calbiochem, Darmstadt, Germany) or 0.15 μg/ml anti-EGFR (Clone: 31G7, Zymed laboratories, Carlsbad, CA, USA) antibodies separately for one hour followed by incubation with HRP-conjugated anti-mouse secondary antibody (Dako REAL Envision Detection System, Dako, Glostrup, Denmark) for 30 minutes and subsequently with DAB chromogen (Dako REAL Envision Detection System, Dako, Glostrup, Denmark) for 3 and 7 minutes for p53 and EGFR, respectively. Sections were counter stained with hematoxylin. The slides were scored by two experienced pathologists blinded for clinical and molecular data. Samples exhibiting nuclear stain in more than 20% tumor epithelium were considered as positive for p53. For EGFR, staining intensity (negative, weak, moderate and strong) and fractional epithelium positivity (≤25%, 25 ≤ 50%, 50 ≤ 75% and 75 ≤ 100%) were scored as 0–3. A summated score greater than 3 was considered as positive.

### DNA isolation

#### From FFPE blocks

8 μM FFPE tissue sections from tumor and matched normal blocks were stained with hematoxylin after deparaffinization. Tumor rich areas identified by the pathologist were scraped off and DNA was isolated using SDS-proteinase K lysis and subsequent phenol-chloroform extraction followed by alcohol precipitation.

#### From frozen tissues

DNA was isolated from fresh resected tumor tissues using the DNeasy Kit (Qiagen, Hamburg, Germany) as per manufacturer’s protocol after confirming **≥**70% neoplastic cellularity.

### *TP53* mutation and human papilloma virus (HPV) screening

Bidirectional sequencing of *TP53* exons 5–8 was carried out on a 3100 Genetic analyzer (ABI inc., Foster city, CA, USA) after PCR amplification using FFPE tumor DNA as template. Primer sequences are given in supplementary Additional file 2: Table S2. Suspected in-dels were confirmed using TA cloning vector (Invitrogen, Carlsbad, CA, USA) as per standard procedure. PCR based screening of HPV was carried out as per standard protocol [7] with GP5^+^ and GP6^+^ primers using DNA isolated from frozen tumor tissue as template. Primer sequences are given in supplementary Additional file 2: Table S2.

### Microsatellite instability (MSI) screening and loss of heterozygosity (LOH) analysis

MSI analysis was performed for the 106 fresh samples using the standard NCI panel of five microsatellites (two mononucleotide repeats *viz.* BAT25 and BAT26 and three dinucleotide repeats *viz.* D2S123, D5S346 and D17S250) using FFPE DNA as template as described earlier [8]. Primer sequences are listed in supplementary Additional file 2: Table S2. Samples were classified as MSI if two or more microsatellites exhibited instability and as microsatellite stable (MSS) if one or none exhibited instability.

LOH analysis was performed (only for fresh samples) based on polymorphic microsatellites located close to putative tongue cancer tumor suppressor genes including tp53CA (*TP53*-17pl3.1), D3S1300 (*FHIT*-3p14.2) and D9S1748 (*CDKN2A*-9p21). LOH status was also assessed for all three dinucleotide microsatellites of the NCI panel namely D2S123 (*hMSH2*-2p15-16), D5S346 (*APC*-5q21) and D17S250 (*BRCA1*-17q11.2). Primer sequences are listed in supplementary Additional file 2: Table S2. Experimental procedure was identical to that of MSI analysis and LOH status was determined as described earlier [7].

### Statistical analysis

Association between clinico-pathological and molecular variables was examined using Fisher’s exact test. Disease specific survival time was calculated as the duration between tumor resection and death. For patients who were lost to follow up or died of reasons other than SCCOT, survival times were censored to the last date on which patients were known to be alive. Kaplan-Meier method was used to estimate survival probability. Log rank test was used to estimate significant differences in survival rates between different groups. Cox proportional hazards model was used to assess the effect of covariates in multivariate analysis.

## Results

**Figure 1:**
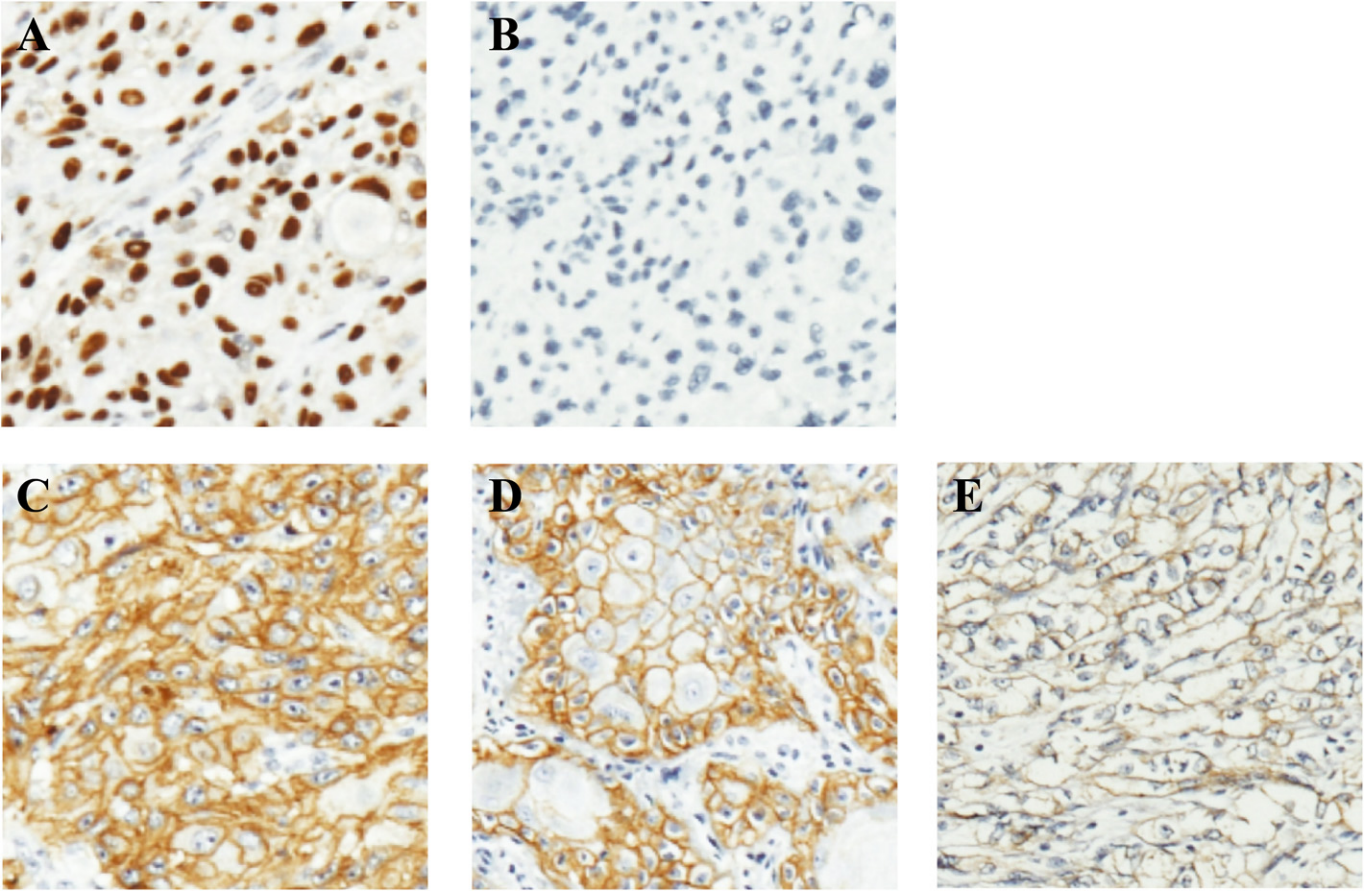
**Immunohistochemistry based detection of p53 and EGFR in primary SCCOT samples.** Representative results of nuclear stabilization **(A)** and negative staining **(B)** of p53 are shown. Panels **C**, **D** and **E** show representative results for strong, moderate and weak EGFR staining, respectively. Original magnification 100x.

Among 121 samples analyzed, 78 (64.46%) exhibited p53 nuclear stabilization (Table 1 and Figure 1A and B). Surprisingly, we observed a significant difference (p = 0.0184) in p53 nuclear staining between young (36/46; 78.26%), and old (42/75; 56%) patients (Table 1). There was no significant association however between p53 stabilization and tobacco use (data not shown). We next screened mutations in exons 5–8 of *TP53* that encode the DNA binding domain and are known to harbor majority of mutations [9]. Mutations (listed in Additional file 3: Table S3), were detected in fifteen of thirty five tumor samples that exhibited p53 nuclear stabilization and in three of twenty six that did not. We did not observe differences in frequency of mutation in young and old patients stratified by p53 nuclear stabilization (5/16, 31.25% in young and 10/19, 52.63% in old among p53 positive tumors; and 0/5, 0% in young and 3/21, 14.28% in older patients among p53 negative tumors). Proportion of transitions, transversions and indels were similar to previous reports for SCCT as per the International Agency for Research on Cancer TP53 database (Additional file 4: Figure S1) and were not significantly different between the two age groups (data not shown). We identified a novel 33 bp deletion, c.616-648del33 (Additional file 5: Figure S2), located in exon 5 in a p53 positive tumor sample obtained from a chronic tobacco chewer that is expected to result in loss of eleven amino acids (143–153). The deleted amino acids include four (143–146) that form part of β-sheet S3 which is important in stabilizing the loop- β sheet- α helix motif, a key domain in formation of p53 DNA binding surface [10]. Majority of p53 positive tumors harboring mutation (12/15) exhibited p53 positivity in greater than 50% tumor cells (Additional file 3: Table S3). In contrast, frequency of mutation was significantly lower (3/14; 21.42%) (Additional file 3: Table S3) in p53 positive tumors exhibiting stabilization in less than 50% cells. In addition, of the three p53 negative tumors that harbored p53 mutation, two exhibited complete absence of staining. Interestingly, missense/inframe mutations were predominantly identified in tumors exhibiting p53 stabilization whereas frameshift mutations resulting in protein truncation were identified exclusively in p53 negative tumors (Additional file 3: Table S3).

**Table 1:** Correlation of p53 nuclear stabilization with patient age

**Age**	**n**	**NS+**	**NS-**	**p-value**
Young (≤45 years)	46	36	10	0.0184
Old (≥46 years)	75	42	33	
Total	121	78	43	

n, Number of samples; NS+, Nuclear stabilization; NS-, Absence of nuclear stabilization;

p value corresponds to Fisher’s exact test.

A significant proportion of HNSCC has been found to express EGFR at high levels [11] and the same was observed in the current study as well (97/121; 80.17%) (Additional file 5: Table S4) (Figure 1C-E). There was no significant difference in EGFR staining in tumors from young and old patients (data not shown). We also analyzed EGFR expression status in matched normal samples for 25 tumors; staining was weak to moderate and was limited to the basal and suprabasal layers (non-keratinized cells). In the corresponding tumors however, strong staining was observed throughout the tumor (data not shown). In addition, in normal epithelium, staining was observed predominantly in cell membrane whereas in tumor cells, cytoplasmic staining was also observed (data not shown).

PCR based screening revealed low proportion of HPV infection (14/106; 13.2%) (Additional file 6: Table S4) and MSI 14/106 (13.2%) (Additional file 7: Figure S3A-E) in our sample cohort. Dinucleotide microsatellites exhibited frequent instability (40/318; 12.58%) compared to mononucleotide microsatellites (13/212; 6.13%) (data not shown). LOH was more frequently observed in *CDKN2A* (28.09%) and *FHIT* (26.37%) than other loci tested (Table 2) (Additional file 7: Figure S3F-G). Nineteen of fifty six samples (33.92%) positive for p53 staining in contrast to only five of thirty five (14.29%) p53 negative samples, exhibited LOH at *FHIT* indicating *FHIT* loss could be a more frequent event in tumors exhibiting p53 nuclear stabilization (p = 0.0508) (Table 3). In addition, this association was stronger (p = 0.0094) in patients with no history of tobacco use (Table 4).

**Table 2:** LOH frequency at different loci

**Microsatellite**	**D2S123/*hMSH2***	**D5S345/*APC***	**D17S143/*BRCA2***	**TP53CA/*TP53***	**D3S1300/*FHIT***	**D9S1748/*CDKN2A***
Informative cases	94	95	90	98	91	89
Frequency of LOH*	2.12 (02)	6.31 (06)	6.67 (06)	11.22 (11)	26.37 (24)	28.09 (25)

LOH, loss of heterozygosity.

*In percentage; Number of samples exhibiting LOH is shown in parenthesis.

**Table 3:** **Correlation of p53 stabilization with *FHIT *** **LOH**

***FHIT *** **status**	**n**	**p53 status**		**p-value**
		**NS+**	**NS-**	
*FHIT* LOH+	24	19	05	0.0508
*FHIT* LOH-	67	37	30	

*FHIT* LOH+, *FHIT* LOH present; *FHIT* LOH-, *FHIT* LOH absent; NS+, p53 Nuclear stabilization; NS-, absence of p53 nuclear stabilization; n, Number of samples; LOH, loss of heterozygosity.

p value corresponds to Fisher’s exact test.

**Table 4:** **Correlation of *FHIT *** **LOH with p53 stabilization and tobacco use**

**P53 status**	**n**	**Tobacco users (50)**		**Tobacco never users (23)**	
		***FHIT *** **LOH+**	***FHIT *** **LOH-**	***FHIT *** **LOH+**	***FHIT *** **LOH-**
NS+	45	09	24	08	4
NS-	28	04	13	01	10
				p value = 0.0094	

*FHIT* LOH+, *FHIT* LOH present; *FHIT* LOH-, *FHIT* LOH absent; NS+, p53 nuclear stabilization; NS-, absence of p53 nuclear stabilization; n, Number of samples; LOH, loss of heterozygosity.

p value corresponds to Fisher’s exact test.

**Figure 2:**
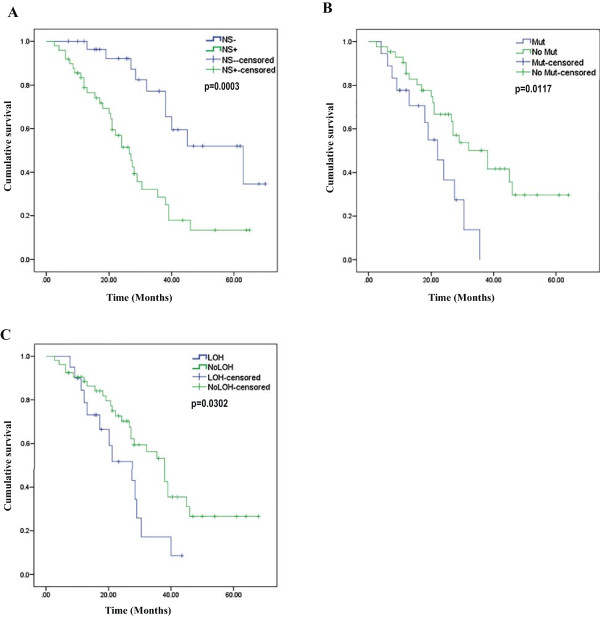
**Kaplan Meier curves of disease specific survival of SCCOT patients based on p53 nuclear stabilization (A), *TP53 *** **mutation (B) and *FHIT *** **LOH status (C).**

Survival data was collected for a total of seventy nine patients; median survival was 30.5 months. Though we did not detect correlation of disease specific survival with pathological stage or grade, there is a significant difference in survival rate between patients with p53 positive and negative tumors (p = 0.0003) (Figure 2A and Table 5). As expected, patients with tumors harboring p53 DNA binding domain mutation were significantly associated with poor survival (p = 0.0117) (Figure 2B and Table 5). *FHIT* loss also exhibited significant effect on disease specific survival (p = 0.0302) (Figure 2C and Table 5) but it was not an independent predictor of worse prognosis, as determined by Cox proportional hazard model.

**Table 5:** **Association of p53 nuclear stabilization and *FHIT *** **loss with disease specific survival of SCCOT patients**

	**Total (n)**	**Dead (n)**	**% Dead**	**Median survival (months)**	**Hazard ratio**^**a**^	**95% CI**	**Significance**^**b**^
Total	79	42	53.16				
**P53 nuclear stabilization**							
NS-	30	10	33.3	63	-	-	0.0003
NS+	49	32	65.31	26.5	3.35.1	1.8293-6.1350	
**P53 mutation**							
Mutation	18	12	66.67	22			0.0117
No mutation	43	22	51.16	38	0.4274	0.1811 to 1.0084	
***FHIT *** **LOH**							
LOH	20	13	65	21	-	-	0.0302
No LOH	53	27	50.94	38	0.4967	0.2265-1.0893	

^a^Hazard ratio was calculated to the first variable in a subgroup (indicated by empty cells).

^b^Corresponds to Log Rank test (Mantel-Cox).

## Discussion

Abrogation of p53 tumor suppressor activity is a frequent event in many cancers, including HNSCC [12]. The frequency of p53 nuclear stabilization identified in SCCOT in the present cohort (64.46%) is in accordance with previous reports from India [13] as well as from the West [14]. Interestingly, frequency of p53 nuclear stabilization was high in young patients (Table 1), suggesting possible role of genetic factors. An earlier study conducted on 724 HNSCC cases reported a similar difference of p53 stabilization between young and older patients [15]. Of interest, a study conducted on aging mice showed a two-fold decline in p53 activity with advancing age, when exposed to radiation [16]. It can perhaps be postulated that age related decline in p53 transcriptional activity may independently contribute to tumorigenesis in old patients perhaps by mimicking mutational inactivation. The distinct occurrence of *TP53* mutation exclusively in samples exhibiting strong or absent p53 immunostain has been observed earlier in ovarian cancer [17]. However, we cannot rule out the possibility of dilution of mutant allele by the wild type allele in samples exhibiting p53 staining in less than 50% cells. Since p53 mutations were also identified in samples not exhibiting nuclear stabilization, using immunostaining alone to identify p53 status may not be an ideal approach. Interestingly, we observed that young patients with p53 nuclear stabilization also exhibited DNA binding domain mutation similar to older SCCOT patients. This is in contrast to a study conducted on SCCOT patients in USA where none of the young patients who exhibited p53 nuclear stabilization harbored mutation [18,19]. There is no previous report of HPV screening performed specifically on SCCOT from India, though few studies on oral squamous cell carcinoma (OSCC) revealed a higher frequency of HPV infection [20], probably due to inclusion of other oral cancer subtypes. Base of tongue squamous cell carcinoma is known to exhibit higher frequency of HPV infection [21].

Previous studies undertaken on HNSCC showed significant variation in MSI (ranging from 1- 65%) across populations, though number of markers analyzed varied significantly [22-24]. Our results suggest the presence of a higher frequency of MSI in SCCOT compared to other HNSCC subtypes, as also reported previously [22]. In this study, dinucleotide microsatellites exhibited frequent instability compared to mononucleotide microsatellites perhaps suggesting the occurrence of a distinct form of instability than the one observed in classical mismatch repair (MMR) deficient tumors [25]. A significant proportion (one-third) of tumors exhibited LOH at D9S1748 (*CDKN2A*) consistent with earlier reports [26]. LOH frequency of D2S123 (*hMSH2*), D5S346 (*APC*) and D17S250 (*BRCA1*) observed in our patient cohort appeared to be lower than previous reports [24]. An earlier report from India revealed marginally higher frequency of LOH at *TP53* locus in oral cancer [27], probably due to influence of tumors other than SCCOT.

*FHIT* harbors one of the most common fragile sites in the genome called FRA3B and is often associated with chromosomal deletions in various cancer cell lines and tumors [28]. P53 inactivation induced genomic instability could be one cause for the association of p53 nuclear stabilization with *FHIT* loss though a similar association with *CDKN2A* LOH was not identified. *FHIT* loss can be expected to be more susceptible to genomic instability given its location within a chromosomal breakpoint region [28]. Strong association of loss of *FHIT* and p53 inactivation in nonsmokers (Table 4) suggests that tumors occurring in tobacco never users with and without p53 inactivation could be distinct entities. Wild type p53 and FHIT are known to have similar roles in inducing apoptosis and cell cycle arrest possibly through Bak and p21 respectively [29]. Therefore, inactivation of *FHIT* and p53 may facilitate tumor cells to evade apoptosis and escape G0/G1 arrest. A recent report suggests that inactivation of both *FHIT* and p53 may have possible synergistic effect resulting in deregulation of proliferation related genes in lung cancer cell lines and tumors [30], particularly in squamous cell carcinoma subtype of non-small cell lung cancer [31]. Ours is however the first study to report such association in SCCOT (Table 3).

To our knowledge, this is the first report to identify p53 inactivation as an independent prognostic marker for poor survival in SCCOT, though it has been reported in HNSCC [32] and OSCC [33]. Few studies have identified *FHIT* to be a predictor of poor survival in OSCC [34] in HNSCC [35]. However, these studies did not analyze the status of p53 aberrations in the tumors. The association of *FHIT* loss with poor survival is probably a result of association with p53 nuclear stabilization.

## Conclusion

Though the study was conducted on a relatively smaller size of samples, it is expected to help in selecting molecular markers for larger studies in the future with more clinical significance. However, this is the most comprehensive molecular genetic study undertaken on Indian SCCOT patients and has identified frequent mutational inactivation of p53 and its significant association with loss of *FHIT*. More importantly, our results show association of wild type p53 and good survival. Genetic aberrations contributing to concomitant *FHIT* loss and p53 stabilization in tumors need to be delineated. It would be interesting to study tumorigenesis pathways contributing to SCCOT in the absence of p53 and *FHIT* inactivation. Given the unique clinico-pathological features associated with SCCOT, this study is an important step towards understanding of this important but hitherto poorly studied HNSCC subtype.

## Abbreviations

SCCT: Squamous cell carcinoma of tongue; SCCOT: Squamous cell carcinoma of oral tongue; HNSCC: Head and neck squamous cell carcinoma; FFPE: Formalin fixed and paraffin embedded; H&E: Hematoxylin and eosin; IHC: Immunohistochemistry; EGFR: Epidermal growth factor receptor; HPV: Human papilloma virus; MSI: Microsatellite instability; LOH: Loss of heterozygosity; MSS: Microsatellite stable; MMR: Mismatch repair; OSCC: Oral squamous cell carcinoma.

## Competing interests

All authors declare that they have no competing interests.

## Authors’ contributions

MDB conceived the study. MDB and RSRA designed the study. RSRA, VK, NAG, SG, SGU, MMA, SD, MR, SR, UKN, MVC acquired data. MDB and RSRA performed statistical analysis. MDB and RSRA prepared manuscript with inputs from all authors. All authors read and approved the final manuscript.

## Pre-publication history

The pre-publication history for this paper can be accessed here:

http://www.biomedcentral.com/1472-6890/14/37/prepub

## Supplementary Material

Additional file 1: Table S1 Clinico-pathological details of SCCOT patients. Click here for file

Additional file 2: Table S2 Primers used in the current study. Click here for file

Additional file 3: Table S3 *TP53* mutations identified in the study. Click here for file

Additional file 4: Figure S1 Frequency of p53 mutation types observed in this study and in International Agency for Research on Cancer (IARC) *TP53* Database. Click here for file

Additional file 5: Figure S2 426-458del33, a novel in-frame deletion identified in *TP53* in SCCOT. Click here for file

Additional file 6: Table S4 Frequency of EGFR expression, HPV infection and MSI. Click here for file

Additional file 7: Figure S3 Representative chromatograms depicting MSI. Click here for file

## References

[B1] Myers JN Elkins T Roberts D Byers RM Squamous cell carcinoma of the tongue in young adults: increasing incidence and factors that predict treatment outcomes Otolaryngol Head Neck Surg 2000 122 44 51 10.1016/S0194-5998(00)70142-2 10629481

[B2] Dahlstrom KR Little JA Zafereo ME Lung M Wei Q Sturgis EM Squamous cell carcinoma of the head and neck in never smoker-never drinkers: a descriptive epidemiologic study Head Neck 2008 30 75 84 10.1002/hed.20664 17694557

[B3] Iype EM Pandey M Mathew A Thomas G Sebastian P Nair MK Oral cancer among patients under the age of 35 years J Postgrad Med 2001 47 171 176 11832617

[B4] Sarkaria JN Harari PM Oral tongue cancer in young adults less than 40 years of age: rationale for aggressive therapy Head Neck 1994 16 107 111 10.1002/hed.2880160202 8021128

[B5] Silverman S Jr Demographics and occurrence of oral and pharyngeal cancers. The outcomes, the trends, the challenge J Am Dent Assoc 2001 132 Suppl 132 Suppl 7S 11S 11803655 10.14219/jada.archive.2001.0382

[B6] Ryott M Wangsa D Heselmeyer-Haddad K Lindholm J Elmberger G Auer G Avall Lundqvist E Ried T Munck-Wikland E EGFR protein overexpression and gene copy number increases in oral tongue squamous cell carcinoma Eur J Cancer 2009 45 1700 1708 10.1016/j.ejca.2009.02.027 19332367 PMC7294540

[B7] Pandilla Ramaswamy KV Gowrishankar S Vamsy CM Patnaik S Uppin S Rao S Kalidindi N Regulagadda S Sundaram C Srinivasulu M Vasala A Bashyam MD Distinct genetic aberrations in oesophageal adeno and squamous carcinoma Eur J Clin Invest 2013 43 1233 1239 10.1111/eci.12163 24102414

[B8] Raman R Kotapalli V Adduri R Gowrishankar S Bashyam L Chaudhary A Vamsy M Patnaik S Srinivasulu M Sastry R Rao S Vasala A Kalidindi N Pollack J Murthy S Bashyam M Evidence for possible non-canonical pathway(s) driven early-onset colorectal cancer in India Mol Carcinog 2014 53 Suppl 1 E181 6 23168910 10.1002/mc.21976 PMC3597761

[B9] Joerger AC Fersht AR Structural biology of the tumor suppressor p53 Annu Rev Biochem 2008 77 557 582 10.1146/annurev.biochem.77.060806.091238 18410249

[B10] Cho Y Gorina S Jeffrey PD Pavletich NP Crystal structure of a p53 tumor suppressor-DNA complex: understanding tumorigenic mutations Science 1994 265 346 355 10.1126/science.8023157 8023157

[B11] Kalyankrishna S Grandis JR Epidermal growth factor receptor biology in head and neck cancer J Clin Oncol 2006 24 2666 2672 10.1200/JCO.2005.04.8306 16763281

[B12] Peltonen JK Helppi HM Paakko P Turpeenniemi-Hujanen T Vahakangas KH P53 in head and neck cancer: functional consequences and environmental implications of TP53 mutations Head Neck Oncol 2010 2 36 10.1186/1758-3284-2-36 21159183 PMC3022569

[B13] Khan Z Tiwari RP Mulherkar R Sah NK Prasad GB Shrivastava BR Bisen PS Detection of survivin and p53 in human oral cancer: correlation with clinicopathologic findings Head Neck 2009 31 1039 1048 10.1002/hed.21071 19340865

[B14] Nylander K Nilsson P Mehle C Roos G p53 mutations, protein expression and cell proliferation in squamous cell carcinomas of the head and neck Br J Cancer 1995 71 826 830 10.1038/bjc.1995.159 7710950 PMC2033757

[B15] De Paula AM Souza LR Farias LC Correa GT Fraga CA Eleuterio NB Silveira AC Santos FB Haikal DS Guimaraes AL Gomez RS Analysis of 724 cases of primary head and neck squamous cell carcinoma (HNSCC) with a focus on young patients and p53 immunolocalization Oral Oncol 2009 45 777 782 10.1016/j.oraloncology.2008.11.015 19359212

[B16] Feng Z Hu W Teresky AK Hernando E Cordon-Cardo C Levine AJ Declining p53 function in the aging process: a possible mechanism for the increased tumor incidence in older populations Proc Natl Acad Sci U S A 2007 104 16633 16638 10.1073/pnas.0708043104 17921246 PMC2034252

[B17] Yemelyanova A Vang R Kshirsagar M Lu D Marks MA Shih Ie M Kurman RJ Immunohistochemical staining patterns of p53 can serve as a surrogate marker for TP53 mutations in ovarian carcinoma: an immunohistochemical and nucleotide sequencing analysis Mod Pathol 2011 24 1248 1253 10.1038/modpathol.2011.85 21552211

[B18] Sorensen DM Lewark TM Haney JL Meyers AD Krause G Franklin WA Absence of p53 mutations in squamous carcinomas of the tongue in nonsmoking and nondrinking patients younger than 40 years Arch Otolaryngol Head Neck Surg 1997 123 503 506 10.1001/archotol.1997.01900050051006 9158397

[B19] Lingen MW Chang KW McMurray SJ Solt DB Kies MS Mittal BB Haines GK Pelzer HJ Overexpression of p53 in squamous cell carcinoma of the tongue in young patients with no known risk factors is not associated with mutations in exons 5–9 Head Neck 2000 22 328 335 10.1002/1097-0347(200007)22:4<328::AID-HED3>3.0.CO;2-R 10862014

[B20] D’Costa J Saranath D Dedhia P Sanghvi V Mehta AR Detection of HPV-16 genome in human oral cancers and potentially malignant lesions from India Oral Oncol 1998 34 413 420 10.1016/S1368-8375(98)00028-1 9861351

[B21] Marur S D’Souza G Westra WH Forastiere AA HPV-associated head and neck cancer: a virus-related cancer epidemic Lancet Oncol 2010 11 781 789 10.1016/S1470-2045(10)70017-6 20451455 PMC5242182

[B22] Wang Y Irish J MacMillan C Brown D Xuan Y Boyington C Gullane P Kamel-Reid S High frequency of microsatellite instability in young patients with head-and-neck squamous-cell carcinoma: lack of involvement of the mismatch repair genes hMLH1 AND hMSH2 Int J Cancer 2001 93 353 360 10.1002/ijc.1337 11433399

[B23] Glavac D Volavsek M Potocnik U Ravnik-Glavac M Gale N Low microsatellite instability and high loss of heterozygosity rates indicate dominant role of the suppressor pathway in squamous cell carcinoma of head and neck and loss of heterozygosity of 11q14.3 correlates with tumor grade Cancer Genet Cytogenet 2003 146 27 32 10.1016/S0165-4608(03)00109-2 14499693

[B24] Koy S Plaschke J Luksch H Friedrich K Kuhlisch E Eckelt U Martinez R Microsatellite instability and loss of heterozygosity in squamous cell carcinoma of the head and neck Head Neck 2008 30 1105 1113 10.1002/hed.20857 18615731

[B25] Loukola A Eklin K Laiho P Salovaara R Kristo P Jarvinen H Mecklin JP Launonen V Aaltonen LA Microsatellite marker analysis in screening for hereditary nonpolyposis colorectal cancer (HNPCC) Cancer Res 2001 61 4545 4549 11389088

[B26] Partridge M Emilion G Pateromichelakis S Phillips E Langdon J Location of candidate tumour suppressor gene loci at chromosomes 3p, 8p and 9p for oral squamous cell carcinomas Int J Cancer 1999 83 318 325 10.1002/(SICI)1097-0215(19991029)83:3<318::AID-IJC6>3.0.CO;2-V 10495423

[B27] Saranath D Tandle AT Deo MG Mehta AR Sanghvi V Loss of p53 gene as a biomarker of high risk oral leukoplakias Indian J Biochem Biophys 1997 34 266 273 9425746

[B28] Croce CM Sozzi G Huebner K Role of *FHIT* in human cancer J Clin Oncol 1999 17 1618 1624 10334551 10.1200/JCO.1999.17.5.1618

[B29] Sard L Accornero P Tornielli S Delia D Bunone G Campiglio M Colombo MP Gramegna M Croce CM Pierotti MA Sozzi G The tumor-suppressor gene *FHIT* is involved in the regulation of apoptosis and in cell cycle control Proc Natl Acad Sci U S A 1999 96 8489 8492 10.1073/pnas.96.15.8489 10411902 PMC17543

[B30] Andriani F Roz E Caserini R Conte D Pastorino U Sozzi G Roz L Inactivation of both *FHIT* and p53 cooperate in deregulating proliferation-related pathways in lung cancer J Thorac Oncol 2012 7 631 642 10.1097/JTO.0b013e318244aed0 22425911

[B31] Lee YC Wu CT Shih JY Jou YS Chang YL Frequent allelic deletion at the *FHIT* locus associated with p53 overexpression in squamous cell carcinoma subtype of Taiwanese non-small-cell lung cancers Br J Cancer 2004 90 2378 2383 15150628 10.1038/sj.bjc.6601778 PMC2409530

[B32] Mannarini L Bertino G Morbini P Villa C Benazzo M Markers of chemoradiation resistance in patients with locally advanced head and neck squamous cell carcinoma, treated by intra-arterial carboplatin and concurrent radiation Acta Otorhinolaryngol Ital 2007 27 173 180 17957847 PMC2640025

[B33] Perrone F Bossi P Cortelazzi B Locati L Quattrone P Pierotti MA Pilotti S Licitra L TP53 mutations and pathologic complete response to neoadjuvant cisplatin and fluorouracil chemotherapy in resected oral cavity squamous cell carcinoma J Clin Oncol 2010 28 761 766 10.1200/JCO.2009.22.4170 20048189

[B34] Kujan O Oliver R Roz L Sozzi G Ribeiro N Woodwards R Thakker N Sloan P Fragile histidine triad expression in oral squamous cell carcinoma and precursor lesions Clin Can Res 2006 12 6723 6729 10.1158/1078-0432.CCR-06-1475 17121892

[B35] Tai SK Lee JI Ang KK El-Naggar AK Hassan KA Liu D Lee JJ Ren H Hong WK Mao L Loss of *FHIT* expression in head and neck squamous cell carcinoma and its potential clinical implication Clin Can Res 2004 10 5554 5557 10.1158/1078-0432.CCR-04-0208 15328196

