# Supplementary material for: P53 nuclear stabilization is associated with FHIT loss and younger age of onset in squamous cell carcinoma of oral tongue
Source: BMC Clin Pathol. 2014 Aug 9;14:37. doi: 10.1186/1472-6890-14-37 (PMC4141988; doi:10.1186/1472-6890-14-37)
Supplement: Additional file 1: Table S1 — Clinico-pathological details of SCCOT patients. [file 1472-6890-14-37-S1.doc]

**Table S1: Clinico-pathological details of SCCOT patients**

| Variable^*^ | Group | n |
| --- | --- | --- |
|  | | |
| Age (121) | Young (≤45 years) | 46 |
|  | Old (≥46 years) | 75 |
|  | | |
| Gender (121) | Male | 85 |
|  | Female | 36 |
|  | | |
| Tobacco use (93) | Never users | 65 |
|  | Users | 28 |
|  | | |
| Alcohol use (86) | Never users | 42 |
|  | Users | 44 |
|  | | |
| Grade (121) | Well differentiated | 95 |
|  | Moderately differentiated | 19 |
|  | Poorly differentiated | 07 |
|  | | |
| Tumor stage (91) | T1 | 44 |
|  | T2 | 36 |
|  | T3 | 11 |
|  | | |
| Node stage (73) | N0 | 40 |
|  | N1 | 14 |
|  | N2 | 19 |

^*^, Number of samples for which information was available is given in parenthesis.

n, Number of samples.
