# Supplementary material for: P53 nuclear stabilization is associated with FHIT loss and younger age of onset in squamous cell carcinoma of oral tongue
Source: BMC Clin Pathol. 2014 Aug 9;14:37. doi: 10.1186/1472-6890-14-37 (PMC4141988; doi:10.1186/1472-6890-14-37)
Supplement: Additional file 2: Table S2 — Primers used in the current study. [file 1472-6890-14-37-S2.doc]

**Table S2: Primers used in the current study**

| Application | Primer | | Sequence (5*’—*3*’*) | 5*’* Modification | |
| --- | --- | --- | --- | --- | --- |
| MSI^*^ & LOH^†^ | BAT25 | F | TCGCCTCCAAGAATGTAAGT | TAMRA | |
|  |  | R | TCTGGATTTTAACTATGGCTC | - | |
|  | BAT26 | F | TGACTACTTTTGACTTCAGCC | 6-FAM | |
|  |  | R | AACCATTCAACATTTTTAACC | - | |
|  | D2S123 | F | AAACAGGATGCCTGCCTTTA | 6-FAM | |
|  |  | R | GGACTTTCCACCTATGGGAC | - | |
|  | D5S346 | F | ACTCACTCTAGTGATAAATCGGG | JOE | |
|  |  | R | AGCAGATAAGACAAGTATTACTAG | - | |
|  | D17S250 | F | GGAAGAATCAAATAGACAAT | 6-FAM | |
|  |  | R | GCTGGCCATATATATATTTAAACC | - | |
|  | TP53CA | F | AGGGATACTATTCAGCCCGAGGTG | 6-FAM | |
|  |  | R | ACTGCCACTCCTTGCCCCATT | - | |
|  | D3S1300 | F | AGCTCACATTCTAGTCAGCCT | JOE | |
|  |  | R | GCCAATTCCCCAGATG | - | |
|  | D9S1748 | F | CACCTCAGAAGTCAGTGAGT | JOE | |
|  |  | R | GTGCTTGAAATACACCTTTCC | - | |
|  | | | | |  |
| *TP53* sequencing | Exon 5 | F | TCAACTCTGTCTCCTTCCTCTT | - | |
|  |  | R | AACCAGCCCTGTCGTCTCTC | - | |
|  | Exon 6 | F | CTCTGATTCCTCACTGATTGCTCT | - | |
|  |  | R | CCACTGACAACCACCCTTAACC | - | |
|  | Exon 7 | F | GCACTGGCCTCATCTTGG | - | |
|  |  | R | GGGTCAGAGGCAAGCAGA | - | |
|  | Exon 8 | F | CTGCCTCTTGCTTCTCTTTTCCTA | - | |
|  |  | R | ATAACTGCACCCTTGGTCTCCTC | - | |
|  | | | | | |
| HPV^‡^ screening | GP5^+^ | | TTTGTTACTGTGGTAGATACTAC | - | |
|  | GP6^+^ | | GAAAAATAAACTGTAAATCATATTC | - | |

^*^, Microsatellite instability; ^†^, Loss of Heterozygosity; ^‡^, Human papilloma virus
