# Supplementary material for: P53 nuclear stabilization is associated with FHIT loss and younger age of onset in squamous cell carcinoma of oral tongue
Source: BMC Clin Pathol. 2014 Aug 9;14:37. doi: 10.1186/1472-6890-14-37 (PMC4141988; doi:10.1186/1472-6890-14-37)
Supplement: Additional file 3: Table S3 — TP53 mutations identified in the study. [file 1472-6890-14-37-S3.doc]

**Table S3. *TP53*** mutations identified in the study.

| Sample ID | P53 IHC  Status^*^ | Exon/Intron | Mutation | | Mutation type | |
| --- | --- | --- | --- | --- | --- | --- |
|  |  |  | DNA | Protein | DNA | Protein |
| 0701 | NS+(45) | 6 | c.647T>C | V216A | Transition | Missense |
| 383 | NS+(90) | 8 | c.844C>T | R282W | Transition | Missense |
| 451 | NS+(75) | 8 | c.839G>C | R280T | Transversion | Missense |
| 419 | NS+(70) | 8 | c.872A>G | K291R | Transition | Missense |
| 439 | NS+(80) | 8 | c.832C>T | P278S | Transition | Missense |
| 445 | NS+(90) | 8 | c.844C>G | R282G | Transversion | Missense |
| 365 | NS+(75) | 6 | c.644G>T | S215I | Transversion | Missense |
| 387 | NS+(55) | 6 | c.590T>A | V197E | Transversion | Missense |
| 391 | NS+(50) | 5 | c.524G>A | R175H | Transition | Missense |
| 481 | NS+(80) | 5 | c.396G>C | K132N | Transversion | Missense |
| 477 | NS+(50) | 6 | c.587G>C | R196P | Transversion | Missense |
| 483 | NS+(70) | 5 | c.523C>T | R175C | Transition | Missense |
| 467 | NS+(25) | 8 | c.818G>A | R273H | Transition | Missense |
| 487 | NS+ (40) | 5 | c.550insA |  | Insertion | Frameshift |
| 385 | NS+(70) | 5 | c.426-458del33 |  | Deletion^†^ | In-frame deletion |
| 393 | NS- (0) | 5 | c.570delC |  | Deletion | Frameshift |
| 397 | NS-(0) | 5+IVS5 | c.548-559, IVS5 +1-16del28 |  | Deletion | Frameshift |
| 417 | NS-(20) | 8 | c.826G>A | E286K | Transition | Missense |

NS+, nuclear stabilization; NS-, absence of nuclear stabilization.

^*^Percentage epithelium exhibiting p53 stabilization is given in parenthesis.

^†^Novel deletion resulting in loss of 11 amino acid residues (143-153) in p53.
