# Supplementary figures and images for: P53 nuclear stabilization is associated with FHIT loss and younger age of onset in squamous cell carcinoma of oral tongue
Source: BMC Clin Pathol. 2014 Aug 9;14:37. doi: 10.1186/1472-6890-14-37 (PMC4141988; doi:10.1186/1472-6890-14-37)

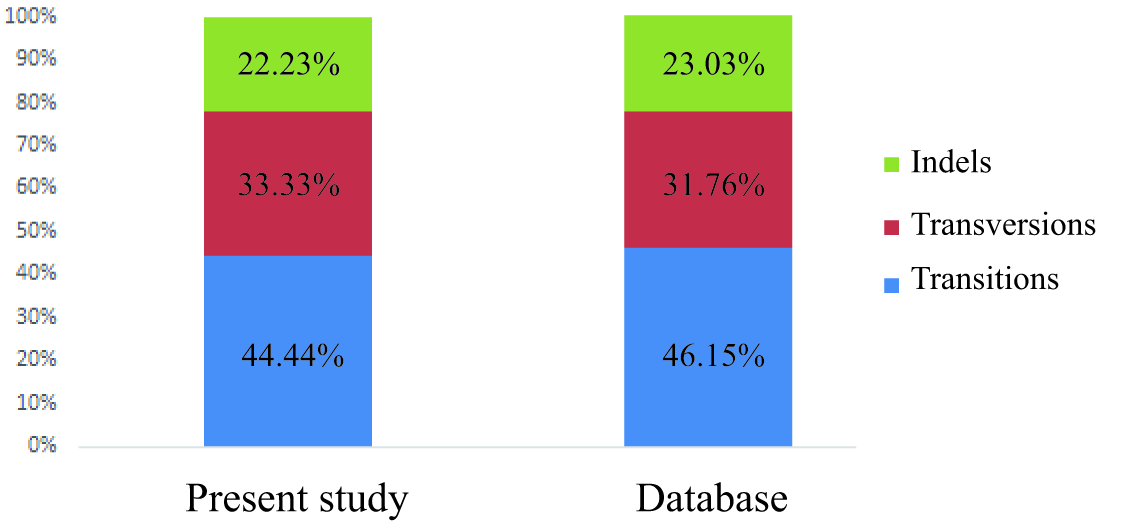

Supplement: Additional file 4: Figure S1 — Frequency of p53 mutation types observed in this study and in International Agency for Research on Cancer (IARC) TP53 Database. [file 1472-6890-14-37-S4.tiff]

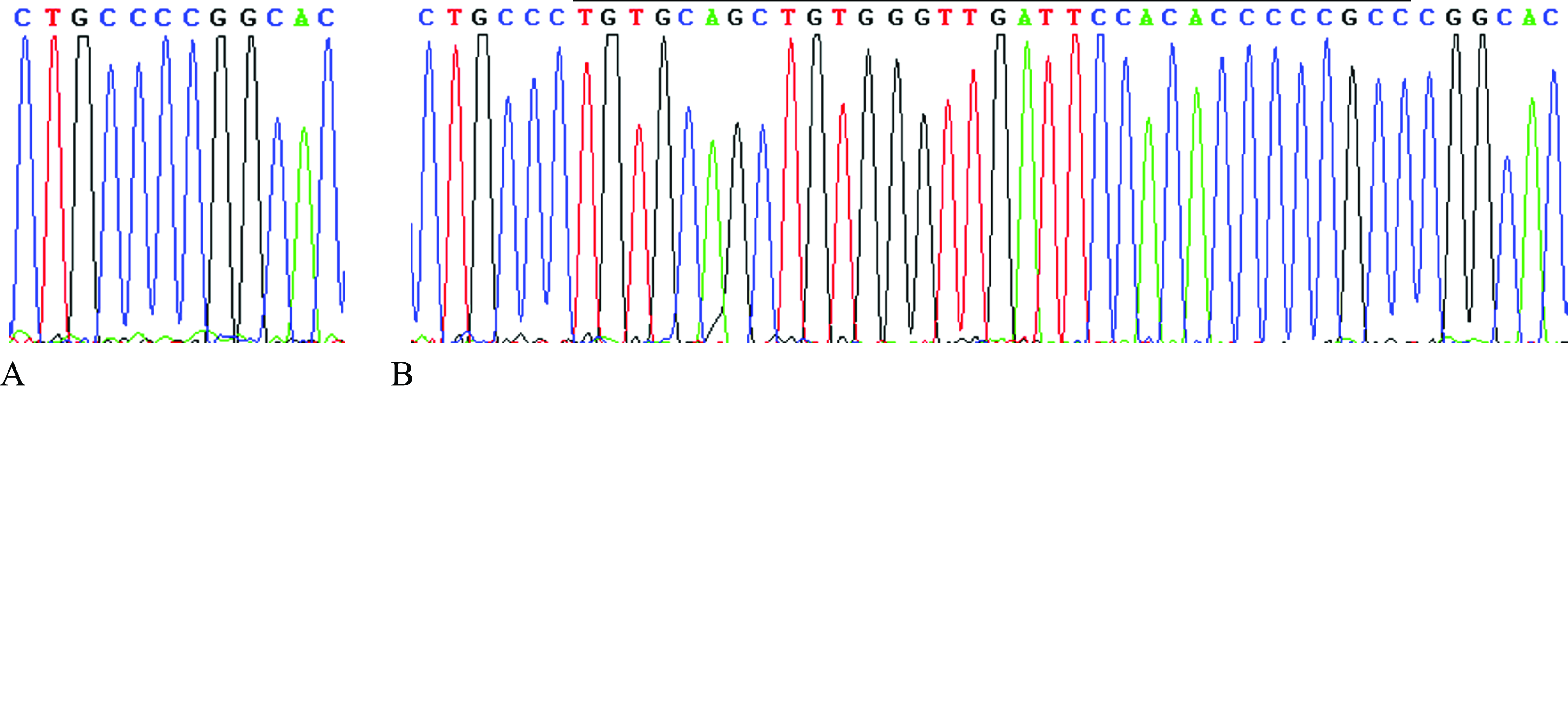

Supplement: Additional file 5: Figure S2 — 426-458del33, a novel in-frame deletion identified in TP53 in SCCOT. [file 1472-6890-14-37-S5.tiff]

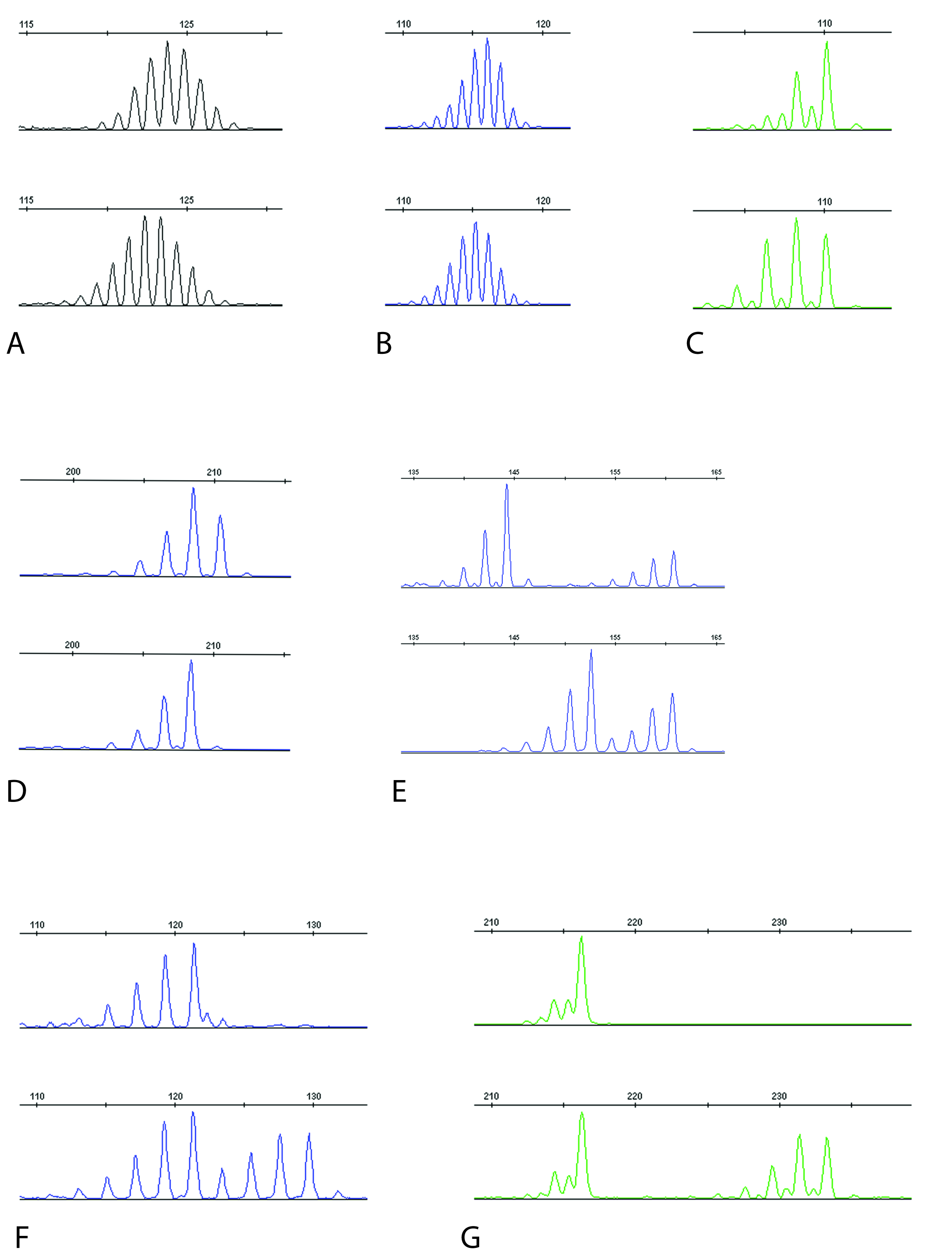

Supplement: Additional file 7: Figure S3 — Representative chromatograms depicting MSI. [file 1472-6890-14-37-S7.tiff]
