# Supplementary material for: P53 nuclear stabilization is associated with FHIT loss and younger age of onset in squamous cell carcinoma of oral tongue
Source: BMC Clin Pathol. 2014 Aug 9;14:37. doi: 10.1186/1472-6890-14-37 (PMC4141988; doi:10.1186/1472-6890-14-37)
Supplement: Additional file 6: Table S4 — Frequency of EGFR expression, HPV infection and MSI. [file 1472-6890-14-37-S6.doc]

Table S4. Frequency of EGFR expression, HPV infection and MSI.

|  | EGFR | | HPV | | MSI | |
| --- | --- | --- | --- | --- | --- | --- |
|  | Positive | Negative | Positive | Negative | Instable | Stable |
| Frequency^*^ | 80.16 (97) | 19.84 (24) | 13.20 (14) | 86.79 (92) | 13.20 (14) | 86.79 (92) |
| N | 121 | | 106 | | 106 | |

EGFR, EGFR expression by immunohistochemistry; HPV, Human papilloma virus infection; MSI, microsatellite instability; N, total number of samples analyzed.

^*^In percentage; number of samples is shown in parenthesis.
